# Overall survival of patients with hepatocellular carcinoma treated with sintilimab and disease outcome after treatment discontinuation

**DOI:** 10.1186/s12885-023-11485-y

**Published:** 2023-10-23

**Authors:** Kang Wang, Yan-Jun Xiang, Hong-Ming Yu, Yu-Qiang Cheng, Jin-Kai Feng, Zong-Han Liu, Yun-Feng Shan, Yi-Tao Zheng, Qian-Zhi Ni, Shu-Qun Cheng

**Affiliations:** 1 Tongji University Cancer Center, Shanghai Tenth People’s Hospital, School of Medicine, Tongji University, Shanghai, 200072 China; 2 Department of Hepatic Surgery VI, Eastern Hepatobiliary Surgery Hospital, Naval Medical University, Shanghai, 200433 China; 3 Department of Hepatobiliary Surgery, The First Affiliated Hospital, Wenzhou Medical University, Wenzhou, 325000 China; 4 CAS Key Laboratory of Nutrition, Metabolism and Food Safety, Shanghai Institute of Nutrition and Health, University of Chinese Academy of Sciences, Chinese Academy of Sciences, Shanghai, 200031 China; 5 Yueyang Hospital of Integrated Traditional Chinese and Western Medicine, Shanghai University of Traditional Chinese Medicine, Shanghai, 200083 China

**Keywords:** Hepatocellular carcinoma, Survival, Clinical observations, Liver cancer

## Abstract

**Background:**

The use of Anti-PD-1 therapy has yielded promising outcomes in hepatocellular carcinoma (HCC). However, limited research has been conducted on the overall survival (OS) of patients with varying tumor responses and treatment duration.

**Methods:**

This retrospective study analyzed HCC patients who received sintilimab between January 2019 and December 2020 at four centers in China. The evaluation of tumor progression was based on Response Evaluation Criteria in Solid Tumors version 1.1. The study investigated the correlation between tumor response and OS, and the impact of drug use on OS following progressive disease (PD).

**Results:**

Out of 441 treated patients, 159 patients satisfied the inclusion criteria. Among them, 77 patients with disease control exhibited a significantly longer OS compared to the 82 patients with PD (median OS 26.0 vs. 11.3 months, *P* < 0.001). Additionally, the OS of patients with objective response (OR) was better than that of patients with stable disease (*P* = 0.002). Among the 47 patients with PD who continued taking sintilimab, the OS was better than the 35 patients who discontinued treatment (median OS 11.4 vs. 6.9 months, *P* = 0.042).

**Conclusions:**

In conclusion, the tumor response in HCC patients who received sintilimab affects OS, and patients with PD may benefit from continued use of sintilimab.

**Supplementary Information:**

The online version contains supplementary material available at 10.1186/s12885-023-11485-y.

## Introduction

Hepatocellular carcinoma (HCC) is a major type of primary liver cancer and the third leading cause of cancer-related death worldwide [1]. Immune checkpoint inhibitor therapy, including PD-1 and PD-L1 inhibitors, have shown clinical benefit in various cancers [2–6], and several studies have shown encouraging clinical activity of anti-PD-1 antibodies in previously treated patients with HCC [7, 8]. The combination of PD-1 inhibitors and other systemic or local therapies has also become a major focus [9–12].

As a type of PD-1 inhibitor, interest in the use of sintilimab for treating HCC has been growing in recent years [13], with several clinical trials conducted to evaluate its safety and efficacy. The ORIENT-32 trial evaluated the efficacy and safety of the combination of sintilimab and IBI305 (a biosimilar of bevacizumab) with sorafenib for the treatment of unresectable HCC patients with hepatitis B virus (HBV) infection, and found that the combination significantly improved overall survival and progression-free survival compared to sorafenib alone, with a manageable safety profile [11]. Additionally, the combination of sintilimab with apatinib plus capecitabine or anlotinib also significantly prolonged the overall survival and progression-free survival of advanced HCC patients [14, 15]. Overall, these studies suggest that sintilimab may be a promising therapeutic option for HCC patients.

However, some questions remain about how to rationally use PD-1 inhibitors [16]. On the one hand, whether tumor response, as a short-term prognostic endpoint, reflects the long-term survival of patients who receiving PD-1/PD-L1 inhibitors was investigated in one report including multiple cancers but not HCC [17]. On the other hand, there is also no standard for the duration of use of PD-1 inhibitors. The KEYNOTE-006 trial investigated the duration of treatment with the PD-1 inhibitor pembrolizumab in the treatment of advanced melanoma, by comparing two different treatment periods. The study found that patients who received a fixed 24-month course of treatment had a significantly higher overall survival rate compared to those who received treatment until disease progression or unacceptable toxicity [18]. Another investigation into the optimal treatment duration of PD-1 inhibitors in the treatment of non-small cell lung cancer (NSCLC) is the Checkmate-153 trial. This study compared different treatment times with nivolumab and found that longer treatment durations were associated with improved survival outcomes [19]. In general, the optimal treatment duration for PD-1 inhibitors may vary depending on the specific type of cancer [20–26]. Currently, there is limited research in the field of HCC.

This study aimed to describe the overall survival (OS) of patients with different responses after treatment with sintilimab and to determine whether treatment discontinuation in patients with progressive disease (PD) has an impact on OS.

## Methods

### Patients

A retrospective study of HCC patients received sintilimab therapy between 2019 and 2020 at the Shanghai Tenth People’s Hospital, the Eastern Hepatobiliary Surgery Hospital, the First Affiliated Hospital of Wenzhou Medical University and the Yueyang Hospital of Integrated Traditional Chinese and Western Medicine was performed.

The inclusion criteria were patients (1) with unresectable HCC diagnosed by histology, cytology, or clinical characteristics according the American Association for the Study of Liver Disease criteria, (2) with good liver function (Child–Pugh liver function score < = 7), (3) who had previously been treated and were either intolerant to the treatment, exhibited an incomplete response, or showed radiographic progression of their disease after treatment. The exclusion criteria were (1) use of sintilimab for postoperative adjuvant therapy and (2) history of other cancers.

### Procedures

Sintilimab was administered intravenously, 200 mg every 3 weeks. If low-grade infusion reactions occurred, the dose should be reduced or treatment should be suspended. When symptoms resolved, treatment was resumed with close observation. The specific doses and protocols used were strictly in accordance with the instructions for use.

In patients whose tumors were under control, sintilimab was continued until disease progression or intolerable toxicities occurred. In this retrospective study, we reviewed the medical records of patients with HCC who had received sintilimab. When patients experienced disease progression during the course of sintilimab use, multidisciplinary consultation was documented in the medical records. Patients were informed of the potential benefits and possible risks of continuing sintilimab, and the decision to continue the treatment was based on the documented consultation between the patient and doctors, while ensuring normal liver function.

### Follow-up and evaluation

Patients were followed up every 6 weeks for the first 6 months, every 3 months for the second half year, and every 6 months thereafter. At each follow-up visit, routine physical examination, laboratory blood tests, and abdominal ultrasound or enhanced CT/MRI were performed. The primary endpoint of this study was OS, which was defined as the time from first use of sintilimab to death due to any cause or the last follow-up (August 2, 2021). Assessment of tumor progression was based on Response Evaluation Criteria in Solid Tumors (RECIST) version 1.1. The definition of tumor response is as follows: complete response (CR), in which all target lesions determined by radiographic studies have disappeared; partial response (PR), in which the sum of the diameters of target lesions has decreased by at least 30%, with no new lesions and no progression of non-target lesions; stable disease (SD), in which there is neither sufficient shrinkage to qualify for PR nor sufficient increase to qualify for PD; PD, in which the sum of the diameters of target lesions has increased by at least 20%, or new lesions have appeared; disease control (DC), which encompasses CR, PR, and SD as composite endpoints; objective response (OR), which includes CR and PR as composite endpoints. Best response refers to the most favorable tumor response observed during the course of treatment. Because tumor response assessment at a fixed time point may miss late responders and is affected by the duration of response, we performed an analysis of the time point to best response, which is more appropriate but varies from patient to patient.

### Statistical analyses

All clinical data were analyzed using IBM SPSS Statistics 23 (New York, NY, USA) or R 4.0 software (http://www.r-project.org/). Student’s t test was used to compare continuous variables, and the χ2 test or Fisher exact test was used to compare categorical variables. Survival curves were generated using the Kaplan–Meier method and compared using the log-rank test.

The time of the highest hazard rate of tumor control or progression during anti-PD-1 therapy was calculated by the hazard function. The hazard function has been well characterized in the context of colorectal and breast cancer [27–29]. Patients who did not have an event at time t were analyzed for risk of an event by the hazard function. In contrast, the Kaplan–Meier method identifies the cumulative event risk for the entire cohort at time t [30]. In other words, the hazard function assessed the instantaneous risk. The kernel-smoothing method was used to estimate the hazard rates [31]. *P* < 0.05 was considered to indicate a significant difference.

## Results

### Population description

**Fig. 1:**
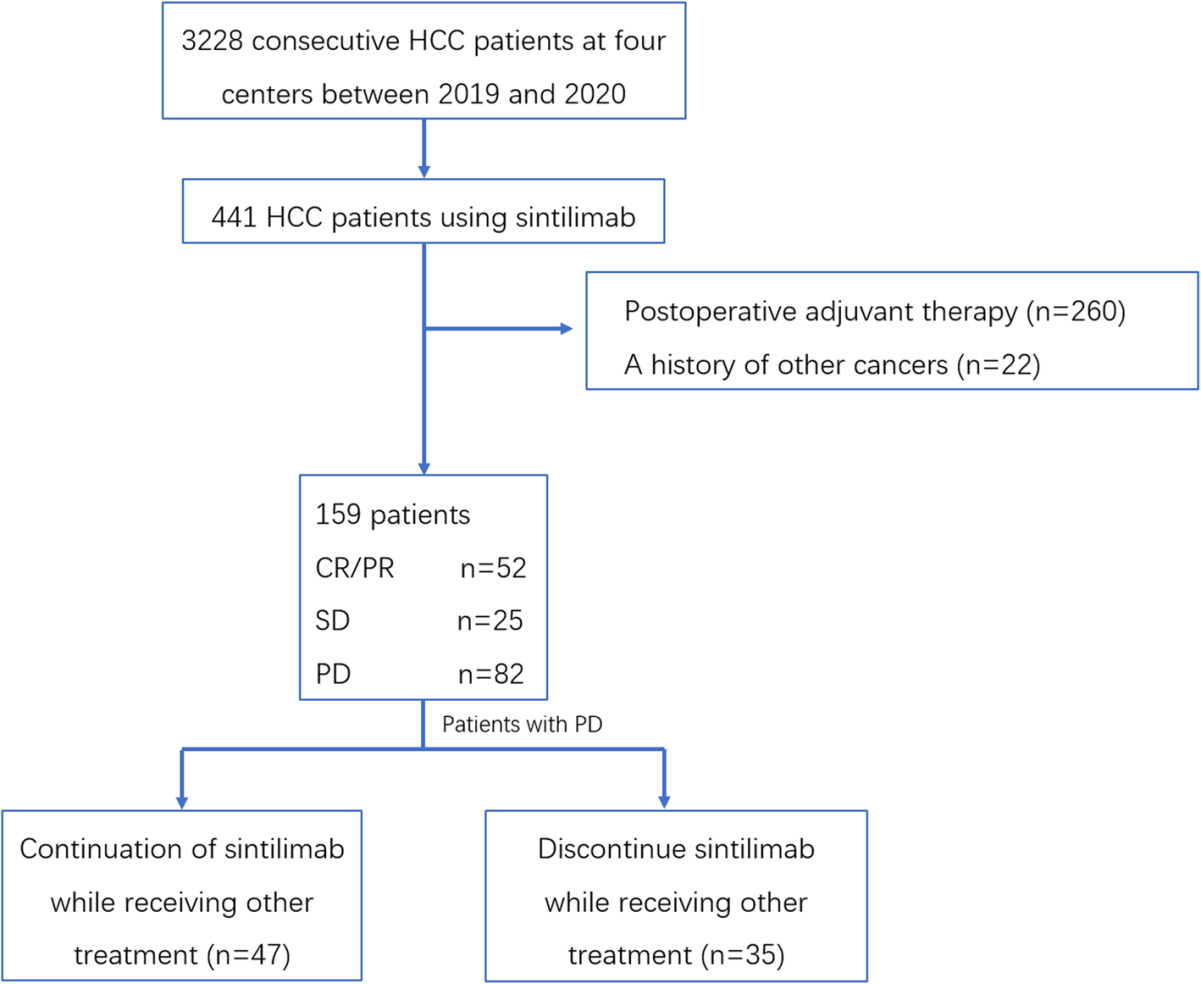
Flow diagram for the present study. HCC hepatocellular carcinoma, CR complete response, PR partial response, SD stable disease, PD progressive disease

From January 2019 to December 2020, 441 patients had been treated with sintilimab in the Eastern Hepatobiliary Surgery Hospital, and among these patients, after exclusions (260 patients received postoperative adjuvant therapy, and 22 patients had a history of other tumors), 159 patients were included in this study (Fig. 1). The patients’ prior therapies are detailed in Supplementary Table 1. During treatment, there were 6 (3.8%) patients with CR, 46 (28.9%) patients with PR, 25 (15.7%) patients with SD, and 82 patients with PD (51.6%). The patient characteristics are detailed in Table 1.

**Table 1:** Demographic and clinical information of the entire patients

Variables	All patients (*n* = 159)
Age (> 65/≤65 years), n (%)	28/131 (17.6/82.4)
Sex (male/female), n (%)	135/24 (84.9/15.1)
HBsAg (positive/negative), n (%)	142/17 (89.3/10.7)
Albumin (> 35/≤35 g/dl), n (%)	125/34 (78.6/21.4)
Total bilirubin (> 17.1/≤17.1 µmol/L), n (%)	79/81 (49.1/50.9)
Prothrombin time (> 13/≤13 s), n (%)	34/125 (21.4/78.6)
Creatinine (> 106/≤106 µmol/L), n (%)	3/156 (1.9/98.1)
Blood glucose (> 7/≤7 mmol/L), n (%)	25/134 (15.7/84.3)
Platelet (> 100/≤100 10^9/L), n (%)	133/26 (83.6/16.4)
ALBI grade (1/2/3), n (%)	87/71/1 (54.7/44.7/0.6)
Alpha fetoprotein (≥ 400/<400 ng/mL), n (%)	74/85 (46.5/53.5)
PIVKA-II (≥ 2050/<2050 mAU/mL), n (%)	68/91 (42.8/57.2)
Liver cirrhosis (yes/no), n (%)	118/41 (74.2/25.8)
Tumor nodules (single/multiple), n (%)	35/124 (22/78)
Maximal tumor diameter (≤ 5/>5 cm), n (%)	30/129 (18.9/81.1)
Extrahepatic metastases (yes/no), n (%)	27/132 (17.0/83.0)
Portal vein tumor thrombus (with/without), n (%)	47/102 (29.6/70.4)
BCLC stage (B/C), n (%)	100/59 (62.9/37.1)
Tumor response (CR/PR/SD/PD), n (%)	6/46/25/82 (3.8/28.9/15.7/51.6)

*Abbreviations: HBsAg *Hepatitis B surface antigen,* ALBI, *Albumin-bilirubin,* PIVKA-II *Protein induced by vitamin K absence or antagonist-II,* BCLC *Barcelona Clinic Liver Cancer,* CR *Complete response,* PR *Partial response*, SD *Stable disease*, PD *Progressive disease

### Overall survival of patients

**Fig. 2:**
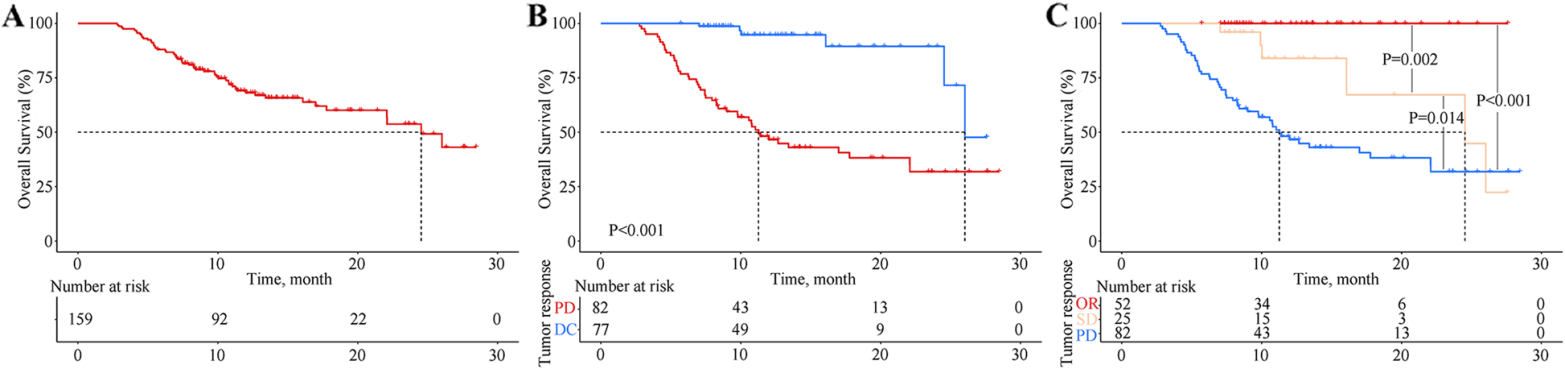
Kaplan-Meier analysis of overall survival. **A** All patients included in the study; **B** patients with disease control and progressive disease; and **C** patients with objective response, stable disease, and progressive disease. PD progressive disease, DC disease control, OR objective response, SD stable disease

The median follow-up of the studied patients was 11 months (range, 3–29 months), with a median OS of 24.5 months (95% CI, 20.6–28.5, Fig. 2A). Among the patients, 77 patients with DC, and the median OS of these patients was 26.0 months (IQR 24.5–26.0), which was significantly higher than the 11.3 months (95% CI 9.1–13.4, *P* < 0.001) of patients with PD (Fig. 2B). There were no deaths among the 52 patients with OR, and the median OS of the 25 patients with SD was 24.5 months (95% CI 8.7–40.4, *P* = 0.002, Fig. 2C).

Multivariate regression analysis showed that direct bilirubin > 17.1 µmol/L, extrahepatic metastases, and DC were independent risk factors for OS in patients treated with sintilimab (Table 2).

**Table 2:** Univariate and multivariate survival analysis in the entire hepatocellular carcinoma cohort

Overall survival	Univariate analysis			Multivariate analysis		
	HR	95% CI	*P* value	HR	95% CI	*P* value
Age (> 65/≤65 years)	1.117	0.545–2.291	0.763			
Sex (male/female)	1.704	0.728–3.986	0.219			
HBsAg (positive/negative)	0.899	0.405–1.993	0.793			
Albumin (> 35/≤35 g/dl)	1.871	1.042–3.360	**0.036**			
Total bilirubin (> 17.1/≤17.1 µmol/L)	2.061	1.184–3.586	**0.011**	1.927	1.102–3.360	**0.021**
Prothrombin time (> 13/≤13 s)	0.740	0.348–1.574	0.434			
Creatinine (> 106/≤106 µmol/L)	1.368	0.188–9.961	0.757			
Blood glucose (> 7/≤7 mmol/L)	1.597	0.822–3.103	0.167			
Platelet (> 100/≤100 10^9/L)	1.255	0.631–2.498	0.518			
Alpha fetoprotein (≥ 400/<400 ng/mL)	1.326	0.774–2.271	0.304			
PIVKA-II (≥ 2050/<2050 mAU/mL)	0.767	0.444–1.326	0.342			
Liver cirrhosis (yes/no)	1.120	0.588–2.133	0.731			
Tumor nodules (single/multiple)	1.549	0.755–3.180	0.233			
Maximal tumor diameter (≤ 5/>5 cm)	1.126	0.575–2.205	0.730			
Extrahepatic metastases (yes/no)	2.057	1.131–3.738	**0.018**	1.925	1.002–3.697	**0.049**
Portal vein tumor thrombus (with/without)	3.070	1.798–5.239	**< 0.001**	2.152	1.197–3.868	**0.010**
Tumor response (DC/PD)	0.115	0.049–0.269	**< 0.001**	0.117	0.050–0.276	**< 0.001**

*Abbreviations: HBsAg *Hepatitis B surface antigen,* PIVKA-II *Protein induced by vitamin K absence or antagonist-II,* DC *Disease control,* PD *Progressive disease

### Hazard rate of tumor response or progression

The median number of sintilimab administrations was not reached in patients who achieved objective tumor response (Supplementary Fig. 1A), and the median number of sintilimab administrations of PD was 9 (95% CI 8.13–9.86, Supplementary Fig. 1B). The hazard function for OR is shown in Supplementary Fig. 1C, and the shape of the OR hazard rate curve over time revealed variation in OR risk. The hazard rate curve of OR patients showed an initial upward trend followed by a downward trend, with the highest hazard rate (0.078) occurring between the third and fourth use of sintilimab, and the hazard rate dropping to zero between the ninth and tenth use. The hazard function for PD is shown in Supplementary Fig. 1D. The risk of patients with PD tended to increase first and then decrease, reaching the highest hazard rate (0.24) between ninth and tenth use.

### Prognosis of patients with PD

During the follow-up period, a total of 82 patients experienced disease progression. Among them, 47 patients continued to receive sintilimab in combination with other treatments, including 30 patients receiving lenvatinib, 13 patients receiving sorafenib, and 4 patients receiving regorafenib. On the other hand, 35 patients discontinued sintilimab but received other treatments, including 21 patients receiving lenvatinib, 10 patients receiving sorafenib, 3 patients receiving regorafenib, and 1 patient abandoning treatment. Baseline characteristics showed no significant differences between the two groups, as shown in Table 3.

**Table 3:** Baseline Characteristics of Patients with Progressive Disease

Characteristics	Continue sintilimab group (*n* = 47)	Discontinue sintilimab group (*n* = 35)	*P* value
Age (year)			0.402
< 65	36 (76.6)	30 (85.7)	
≥ 65	11 (23.4)	5 (14.3)	
Gender			1.000
Female	6 (12.8)	4 (11.4)	
Male	41 (87.2)	31 (88.6)	
HBsAg			0.374
Positive	37 (78.7)	31 (88.6)	
Negative	10 (21.3)	4 (11.4)	
Liver cirrhosis			1.000
Yes	23 (48.9)	18 (51.4)	
No	24 (51.1)	17 (48.6)	
Maximal tumor diameter (cm)			1.000
< 5	12 (25.5)	8 (22.9)	
≥ 5	35 (74.5)	27 (77.1)	
Tumor nodules			0.094
Multiple	41 (87.2)	25 (71.4)	
Single	6 (12.8)	10 (28.6)	
AFP (ng/mL)			0.824
< 400	27 (57.4)	19 (54.3)	
≥ 400	20 (42.6)	16 (45.7)	
PIVKA-II (mAU/mL)			0.498
< 2050	27 (57.4)	23 (65.6)	
≥ 2050	20 (42.6)	12 (34.3)	
TB (umol/L)			0.188
< 17.1	26 (55.3)	14 (40.0)	
≥ 17.1	21 (44.7)	21 (60.0)	
Albumin (g/L)			1.000
< 35	11 (23.4)	9 (25.7)	
≥ 35	36 (76.6)	26 (74.3)	
ALBI grade			0.284
1	31 (66.0)	19 (54,3)	
2	16 (34.0)	16 (45.7)	
3	0 (0.0)	0 (0.0)	
Prothrombin time (second)			0.374
>13	10 (21.3)	4 (11.4)	
≤ 13	27 (78.7)	31 (88.6)	
Creatinine (umol/L)			NA
> 106	0 (0.0)	0 (0.0)	
≤ 106	47 (100.0)	35 (100.0)	
Platelet (X10^9^/L)			0.251
< 100	6 (12.8)	8 (22.9)	
≥ 100	41 (87.2)	27 (77.1)	
Blood glucose (mmol/L)			0.567
< 7	7 (14.9)	7 (20.0)	
≥ 7	40 (85.1)	28 (80.0)	
Extrahepatic metastases			0.580
Yes	10 (21.3)	5 (85.7)	
No	37 (78.7)	30 (14.3)	
Portal vein tumor thrombus			0.418
With	16 (34.0)	14 (40.0)	
Without	31 (66.0)	21 (60.0)	
BCLC stage			0.969
B	28 (59.6)	21 (46.7)	
C	19 (40.4)	14 (53.3)	
Subsequent treatment			0.706
Lenvatinib	30 (63.8)	21 (60.0)	
Sorafenib	13 (29.8)	10 (28.6)	
Regorafenib	4 (8.5)	3 (8.6)	
Abandonment	0 (0.0)	1 (2.9)	

*Abbreviations: HBsAg *Hepatitis B surface antigen,* AFP *Alpha fetoprotein,* TB *Total bilirubin* PIVKA-II *Protein induced by vitamin K absence or antagonist-II,* ALBI *Albumin-bilirubin,* BCLC *Barcelona Clinic Liver Cancer,* TACE *Transarterial chemoembolization

**Fig. 3:**
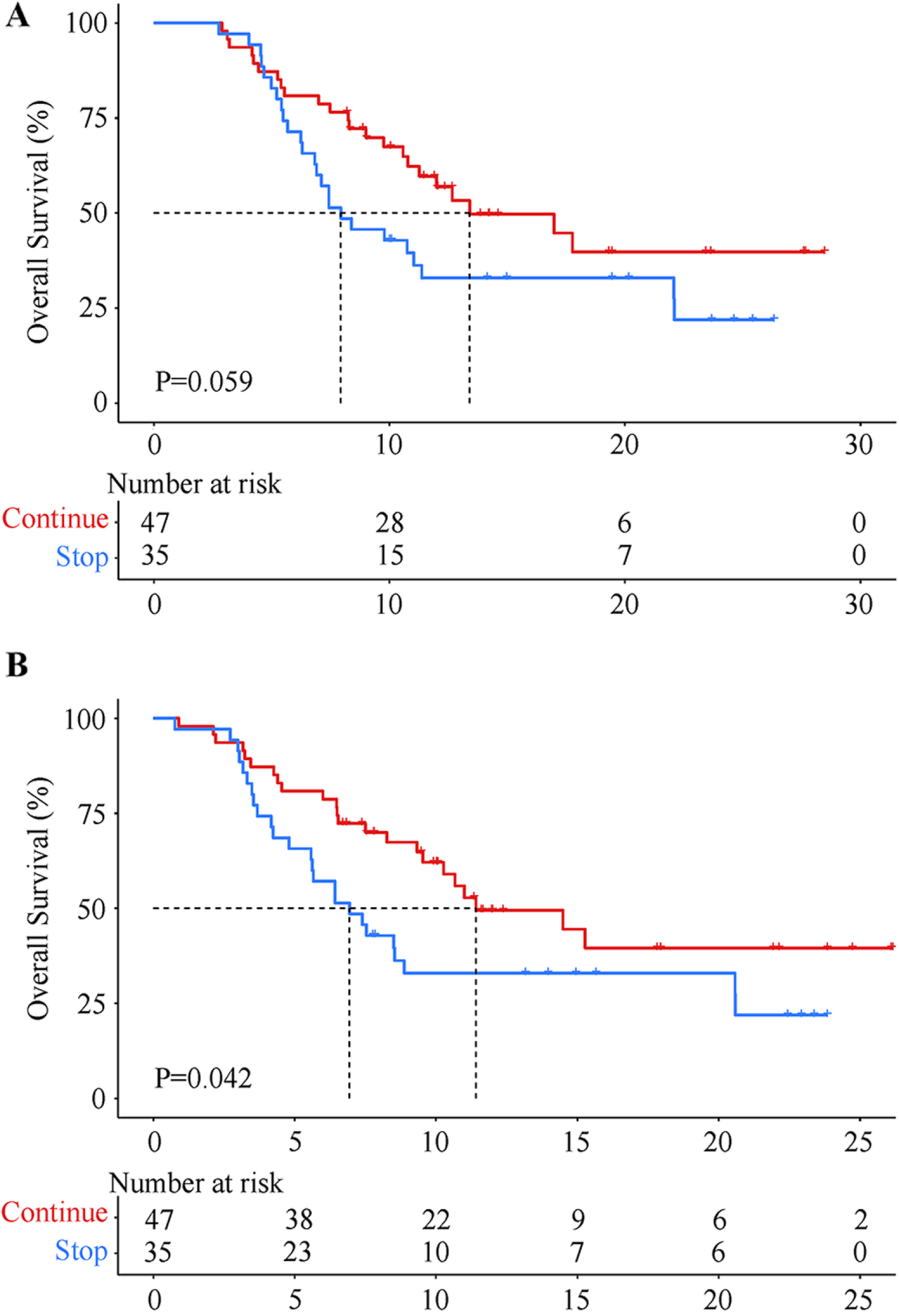
Overall survival in patients who continued and discontinued sintilimab after progressive disease. **A** The starting point was the initiation of sintilimab treatment, while **B** the starting point was the time of diagnosis of progressive disease

In the cohort of patients experiencing disease progression, the discontinuation group had 25 patients who died (18 due to disease progression and 7 due to hepatic failure), while the continuation group had 23 patients who died (15 due to disease progression, 8 due to hepatic failure). When using the start of sintilimab treatment as the starting point, the median OS was 13.4 months (95% CI 6.8–20.0) in the continuation group and 7.9 months (95% CI 4.8–11.0) in the discontinuation group, with no significant difference between the two groups (HR = 0.582, 95% CI 0.330–1.029, *P* = 0.059, Fig. 3A). When setting the starting point as occurrence of PD, the median OS was 11.4 months (95% CI 6.0-16.8) in the continuation group, which was significantly higher than the 6.9 months (95% CI 4.8–9.1) in the discontinuation group (HR = 0.559, 95% CI 0.316–0.987, *P* = 0.042, Fig. 3B).

Regarding safety, as shown in Table 4, the most common treatment emergent adverse event associated with sintilimab of any grade in the continuation group was fatigue (10.6%). The most common grade 3/4 adverse event was elevated aspartate aminotransferase (4.2%).

**Table 4:** Treatment emergent adverse events associated with sintilimab

Adverse Events	Continue sintilimab group (*n* = 47)	
	Any grade, n (%)	Grade 3/4. n (%)
Fatigue	5 (10.6)	0
Pruritus	4 (8.5)	0
Elevated aspartate aminotransferase	4 (8.5)	2 (4.2)
Rash	3 (6.4)	1 (2.1)
Diarrhoea	2 (4.2)	0
Dysphonia	1 (2.1)	0
Nausea	1 (2.1)	0
Weight decreased	1 (2.1)	0
Alopecia	1 (2.1)	0
Decreased appetite	1 (2.1)	0

The hazard function for death is shown in Supplementary Fig. 2. The highest death hazard rate was 0.055 in the continued treatment group, while it was 0.11 in the treatment discontinuation group.

## Discussion

This study has made two main findings. Firstly, tumor response in patients treated with sintilimab was correlated with long-term prognosis, with patients exhibiting a better tumor response having a more favorable prognosis. And we have generated hazard curves for tumor objective response and disease progression. Secondly, patients who experienced disease progression may still benefit from subsequent treatment with sintilimab. The findings of this study may have significant implications for the clinical use of sintilimab and subsequent trial design.

The present international guidelines for the clinical management of HCC suggest the use of systemic therapies for patients with advanced or intermediate stages who progress to chemoembolization [32–34]. However, in the case of HCC, the accuracy of radiological response assessment using RECIST criteria has been questioned due to the finding that some therapies can significantly improve survival despite a very low response rate [35]. The EVOLVE-1 trial evaluated the efficacy of everolimus in treating advanced HCC patients who had previously failed sorafenib treatment. The study found that compared to placebo, everolimus significantly improved DCR in this patient population, but did not improve OS [36]. However, in the recent IMbrave150 trial, patients treated with atezolizumab plus bevacizumab had better ORR and OS compared to Sorafenib [37]. This suggests that the relationship between tumor response and long-term survival may not be constant across different systemic treatments for HCC. In our study, the tumor response of patients receiving sintilimab indeed impacted long-term survival. The overall survival of patients with OR was superior to those with SD and PD. This outcome is in line with previous reports, and given that OR is a favorable prognostic feature, it is not surprising [38]. In daily practice, clinicians often pay more attention to patients with PD and change treatment methods to improve their long-term prognosis. However, the OS of patients with SD was also worse than that of patients with OR, and perhaps more aggressive treatment should be considered to prolong the OS of these patients. According to the hazard rate curve, the highest hazard rate for achieving objective tumor response occurs between the use of the third and fourth doses of sintilimab, indicating a time interval between the initiation of immunotherapy and its benefit [39–41]. The highest risk time for disease progression is between the ninth and tenth use of sintilimab, which may be due to the emergence of acquired resistance [42].

Furthermore, we conducted a subgroup analysis on patients with disease progression. The results indicated that compared to the discontinuation group, the group that continued to receive treatment had a better OS. The discontinuation group had higher rates of progression and peak hazard of death than the group that continued treatment. This may be due to the fact that some patients have a longer response time to sintilimab, or due to a synergistic effect between PD-1 inhibitors and local regional therapy [43–46]. Furthermore, no unexpected adverse events were observed in the group of patients continuing to receive sintilimab. The above evidence suggests that for patients receiving sintilimab, treatment should not be simply discontinued even if tumor progression occurs. The risk of discontinuing anti-PD-1 therapy has also been reported in previous studies, such as in advanced melanoma patients with PR or SD who had a higher risk of disease progression after discontinuation than patients with CR [47], possibly due to discontinuation of PD-1/PD-L1 inhibition. Rapid antibody clearance results in a short on-target treatment lifespan in the local tumor environment [48]. Furthermore, resistance to the target agent may also develop when chronic PD-1/PD-L1 blockade is lifted [49].

The findings of this study may provide guidance for the design of future clinical trials involving sintilimab. Even in the case of disease progression, some patients may still benefit from sintilimab after thorough evaluation. Furthermore, longer follow-up of patients with PD to investigate the mechanisms of the innate immune system and prospective trials aimed at evaluating the duration of treatment for patients after progression (the second randomization of patients with PD) may help confirm our findings. Additionally, further research is needed to determine if timely changes in treatment plans for patients with SD can improve OS.

We must acknowledge the limitations of our study. First, this is a retrospective study with inherent deficiencies, and the PD-L1 expression of the tumor could not be determined. Second, this was a study conducted in China, a hepatitis B virus (HBV)-endemic area, which may have influenced the results. Third, the heterogeneity of patients’ treatment history before receiving sintilimab may have had an impact on the results.

In conclusion, tumor response in HCC patients treated with sintilimab affects the OS, and sintilimab should be used for a fixed period of time to prevent premature discontinuation of therapy due to pseudoprogression. And regular follow-up is necessary for patients receiving long-term sintilimab treatment to prevent adverse outcomes due to drug resistance, and that continued use of sintilimab in combination with other therapies may be beneficial for patients with disease progression but without intolerable toxicity. However, it is important to note that the retrospective nature of the study limits the strength of the conclusions that can be drawn. Additional research, such as prospective studies and randomized controlled trials, are needed to further explore the potential benefits and risks of long-term sintilimab use and determine the optimal treatment strategies for patients with HCC.

### Supplementary Information

**Additional file 1.**

**Additional file 2.**

**Additional file 3.**

## Data Availability

The data that support the findings of this study are available from the corresponding author upon reasonable request.

## Abbreviations

HCC: Hepatocellular carcinoma
PD: Progressive disease
OS: Overall survival
PFS: Progression-free survival
CR: Complete response
PR: Partial response
SD: Stable disease
DC: Disease control
OR: Objective response
