# Supplementary material for: Overall survival of patients with hepatocellular carcinoma treated with sintilimab and disease outcome after treatment discontinuation
Source: BMC Cancer. 2023 Oct 23;23:1017. doi: 10.1186/s12885-023-11485-y (PMC10591394; doi:10.1186/s12885-023-11485-y)
Supplement: Supplementary file 1 — Additional file 1. [file 12885_2023_11485_MOESM1_ESM.docx]

Supplementary table 1. Previous therapies of the studied patients.

| Therapeutic method | All patients (n=159) |
| --- | --- |
| TACE | 77 (48.4) |
| TACE plus sorafenib | 9 (5.7) |
| TACE plus lenvatinib | 40 (25.2) |
| Sorafenib | 2 (1.3) |
| Lenvatinib | 6 (3.8) |
| Radiotherapy | 22 (13.8) |
| Radiotherapy plus lenvatinib | 3 (1.9) |

TACE, trans-arterial chemoembolization.
