# Supplementary figures and images for: Overall survival of patients with hepatocellular carcinoma treated with sintilimab and disease outcome after treatment discontinuation
Source: BMC Cancer. 2023 Oct 23;23:1017. doi: 10.1186/s12885-023-11485-y (PMC10591394; doi:10.1186/s12885-023-11485-y)

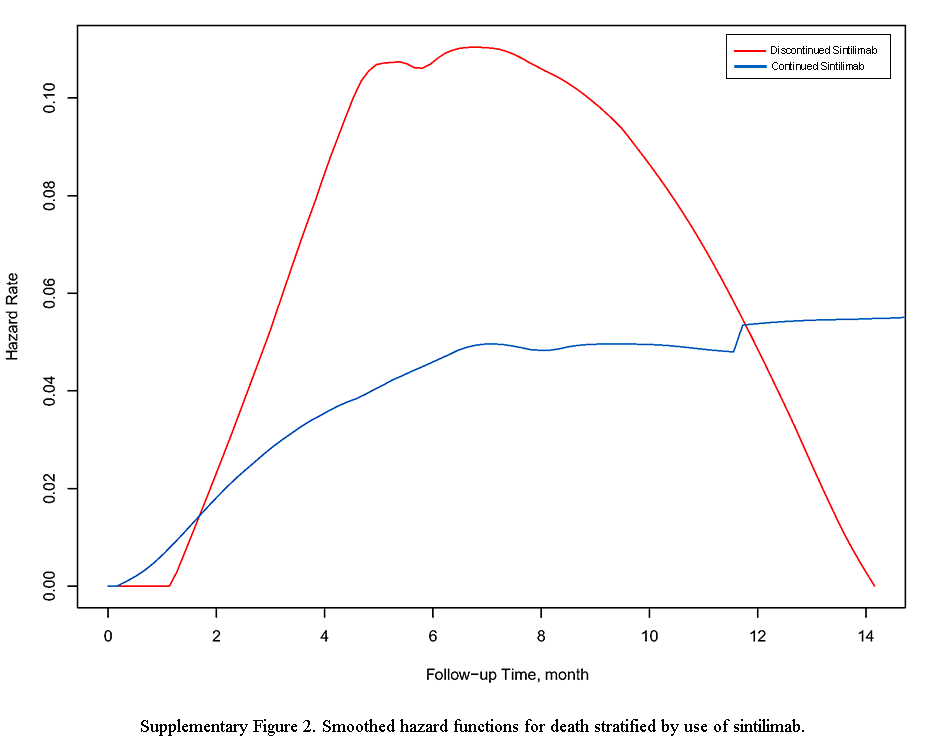

Supplement: Supplementary file 2 — Additional file 2. [file 12885_2023_11485_MOESM2_ESM.png]

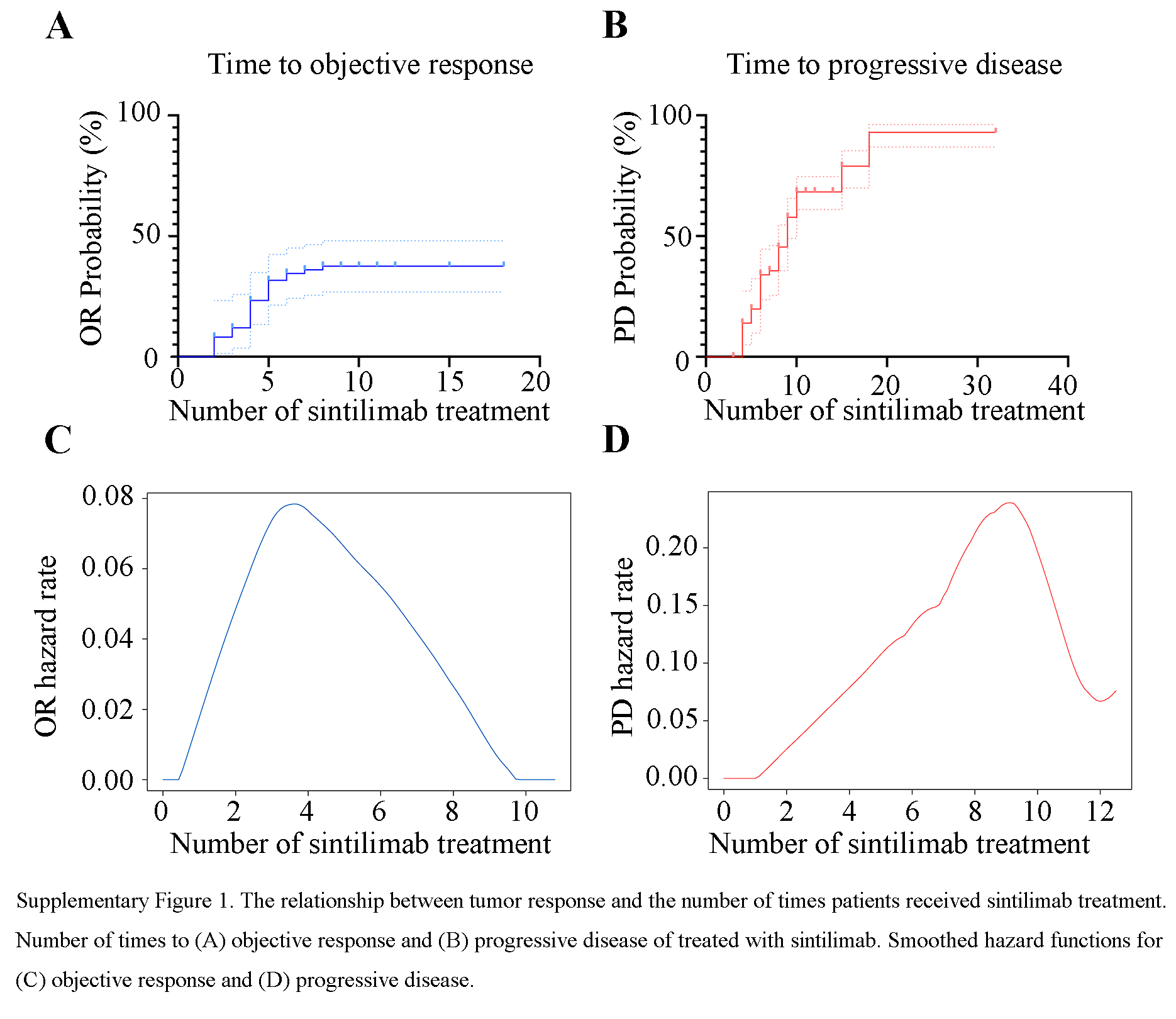

Supplement: Supplementary file 3 — Additional file 3. [file 12885_2023_11485_MOESM3_ESM.png]
